# Supplementary material for: Angstrom-confined catalytic water purification within Co-TiOx laminar membrane nanochannels
Source: Nat Commun. 2022 Jul 11;13:4010. doi: 10.1038/s41467-022-31807-1 (PMC9273791; doi:10.1038/s41467-022-31807-1)
Supplement: Supplementary file 1 — Supplementary Information [file 41467_2022_31807_MOESM1_ESM.pdf]

Supplementary Information

Angstrom-confined catalytic water purification within $Co\text{-}TiO_x$ laminar membrane nanochannels

Chenchen Meng[1,2#], Baofu Ding[3,4#], Shaoze Zhang[5#], Lele Cui[1], Kostya (Ken) Ostrikov[6], Ziyang Huang[4], Bo Yang[2], Jae-Hong Kim[7] and Zhenghua Zhang[1*]

[1]Institute of Environmental Engineering & Nano-Technology, Guangdong Provincial Engineering Research Centre for Urban Water Recycling and Environmental Safety, Tsinghua Shenzhen International Graduate School, Tsinghua University, Shenzhen 518055, China

[2]College of Chemistry and Environmental Engineering, Shenzhen University, Shenzhen 518060, China.

[3]Institute of Technology for Carbon Neutrality/Faculty of Materials Science and Engineering, Shenzhen Institute of Advanced Technology, CAS, Shenzhen, 518055, China.

[4]Tsinghua-Berkeley Shenzhen Institute and Tsinghua Shenzhen International Graduate School, Tsinghua University, Shenzhen 518055, China

[5]National Engineering Laboratory for Vacuum Metallurgy, Engineering Laboratory for Advanced Battery and Materials of Yunnan Province, Kunming University of Science and Technology, Kunming 650093, China.

[6]School of Chemistry and Physics and QUT Centre for Materials Science, Queensland University of Technology (QUT), Brisbane 4000, Australia.

[7]Department of Chemical and Environmental Engineering, Yale University, New Haven, Connecticut 06520-8286, United States.

[#] These authors contributed equally to this work.

[*] Corresponding author: zhenghua.zhang@sz.tsinghua.edu.cn (Z. Zhang)

1. Materials

Reagents and materials

Ranitidine Hydrochloride (> 98%) was obtained from Tokyo Chemical Industry Co. Ltd. PMS ($KHSO_5$, ≥99.0%), titanium dioxide (TiO_2, ≥99.0%), cobalt oxide (CoO, ≥99.0%), potassium carbonate (K_2CO_3, ≥99.0%), tert-butanol (TBA, ≥99.5%), methanol (≥99.5%), *p*-benzoquinone (*p*-BQ, ≥99%), *L*-histidine (*L*-HIS, ≥99.5%), rhodamine B (RhB, IND), methyl orange (MO, IND), methylene blue (MB, AR), tetracycline(TC, AR) and phenol (ACS) were all from Shanghai Aladdin Biochemical Co., Ltd., China. Tetrabutylammonium hydroxide (TBAOH, 40 wt. % in H_2O) and 5, 5-dimethyl-1-pyrroline (DMPO, ≥99.0%) were received from Sigma-Aldrich. 2,2,6,6-tetramenthyl-4-piperidone hydrochloride (TEMP, ≥99.0%) was purchased from Dojindo Co., Ltd. Bisphenol A (BPA, CP) was purchased from Shanghai Meryer Co., Ltd. Carbamazepine (CBZ, ≥98%) was supplied by Shanghai Yuanye Bio-Technology Co., Ltd. Mixed cellulose ester (MCE, 0.22 μm pore size, 45 mm diameter) membrane was provided by Tianjin Jinteng Experimental Equipment Co., Ltd.

2. Results

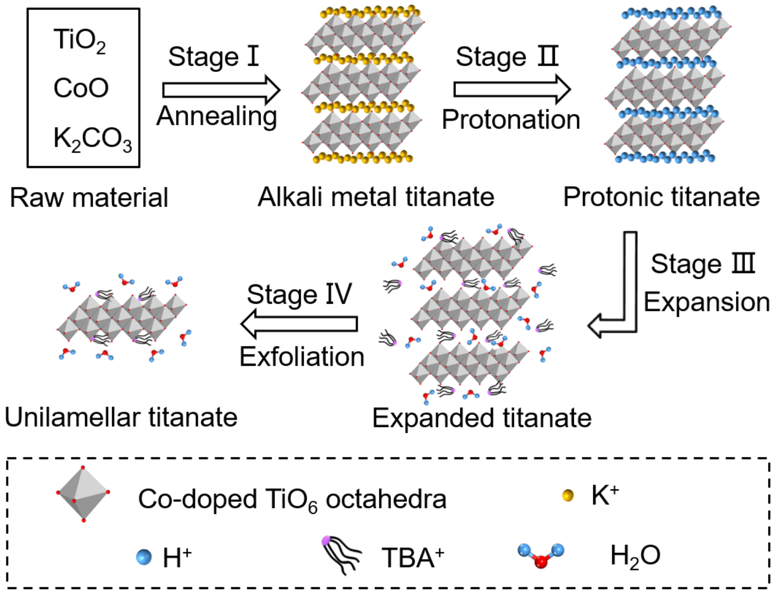

Fig. S1. Fabrication of Co-TiO$_x$ nanosheets. Schematic of Co-TiO$_x$ nanosheets synthesis using a four-stage approach.

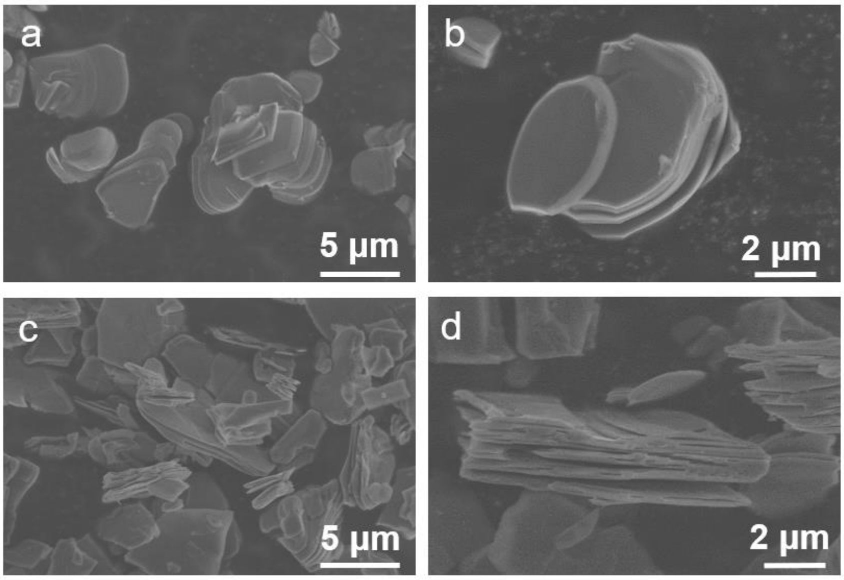

Fig. S2. SEM analysis. Scanning electron microscopy (SEM) images of (a, b) stage-I and (c, d) stage-II products.

A layered structure of the stage-II product with an expanded interlayer spacing compared to the stage-I product can be observed from scanning electron microscope (SEM) images (Fig. S2), which is attributed to the proton exchange with alkali ions.

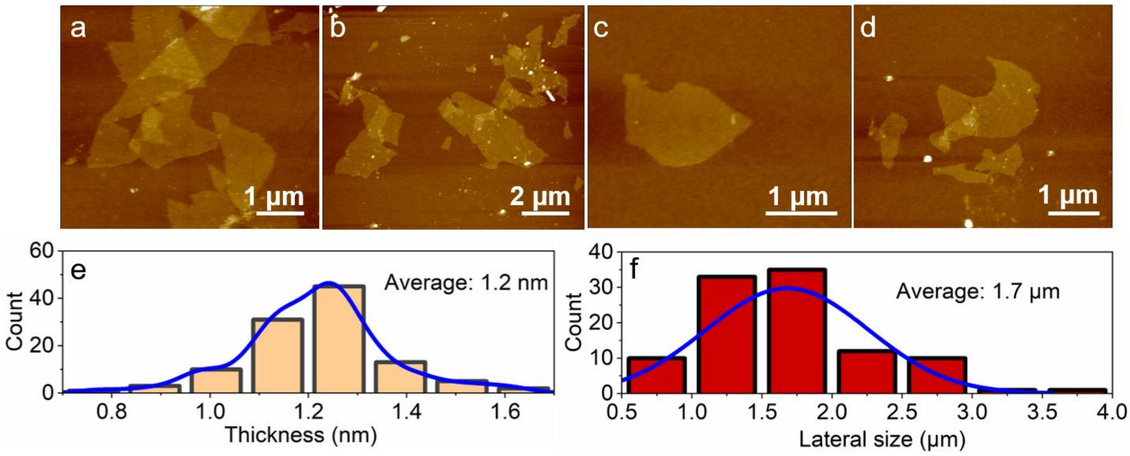

Fig. S3. AFM characterization of Co-TiO$_x$ nanosheets. Atomic force microscopy (AFM) images (a-d) and (e, f) average thickness and lateral size of Co-TiO$_x$ nanosheets.

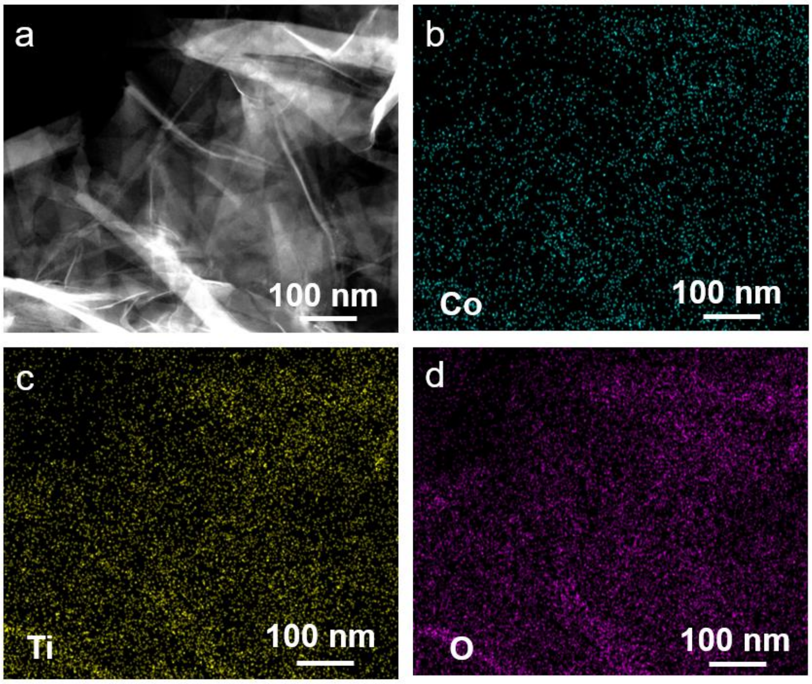

Fig. S4. TEM and EDX analysis of Co-TiO$_x$ nanosheets. (a) Transmission electron microscopy (TEM) image and (b-d) Energy-dispersive X-ray spectroscopy (EDX) mapping images of Co-TiO$_x$ nanosheets.

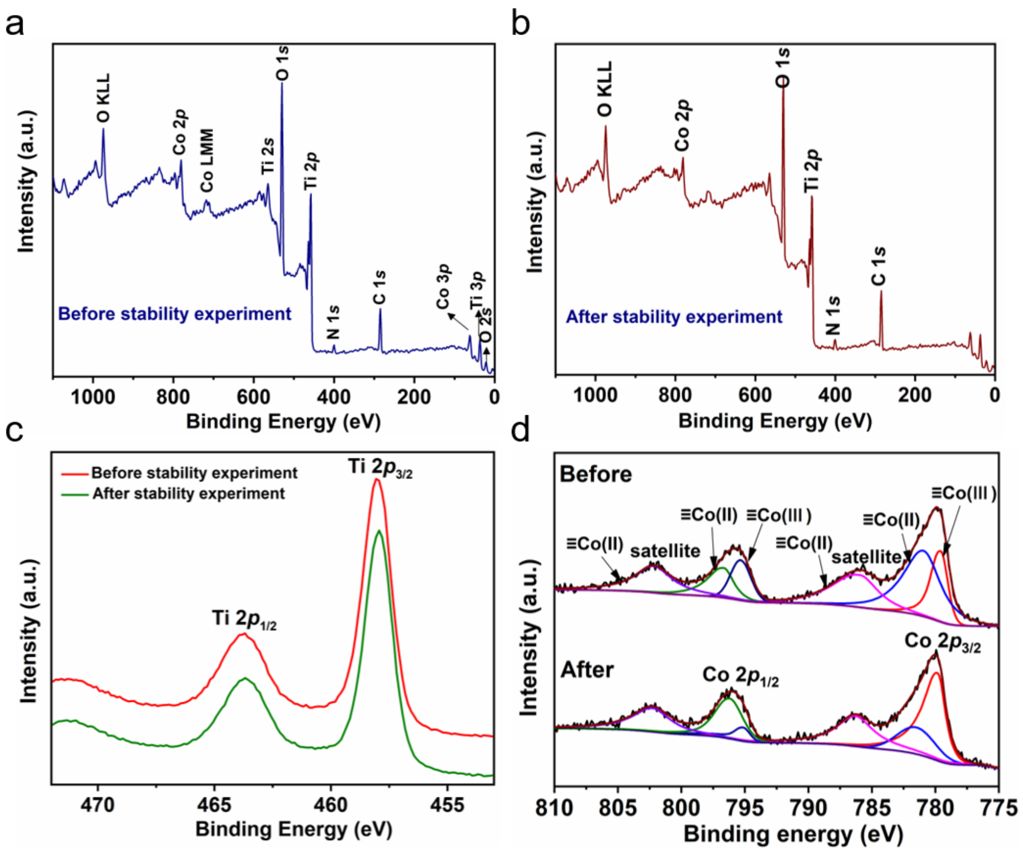

Fig. S5. XPS analysis of Co-TiO$_x$ membranes. XPS survey scan of Co-TiO$_x$ membranes (a) before and (b) after 100 h stability experiment. (c) Ti 2*p* and (d) Co2*p* spectra of Co-TiO$_x$ membranes before and after 100 h stability experiment.

As shown in Figs. S5a and S5b, all peaks assigned to the elements Ti, C, O, N, and Co are the same in Co-TiO$_x$ membranes before and after 100 h stability experiment. The peaks of Ti 2$p_{3/2}$ and Ti 2$p_{1/2}$ (Fig. S5c) for both samples are located at 458.02 eV and 463.76 eV corresponding to ≡Ti(IV). It proves that ≡Ti(IV) does not participate in the catalytic reaction. The peaks (Fig. S5d) with binding energies of 781.0, 786.0, 796.8, and 802.2 eV are assigned to ≡Co(II), and the peaks located at 780.0 eV and 795.3 eV represent ≡Co(III)[1].

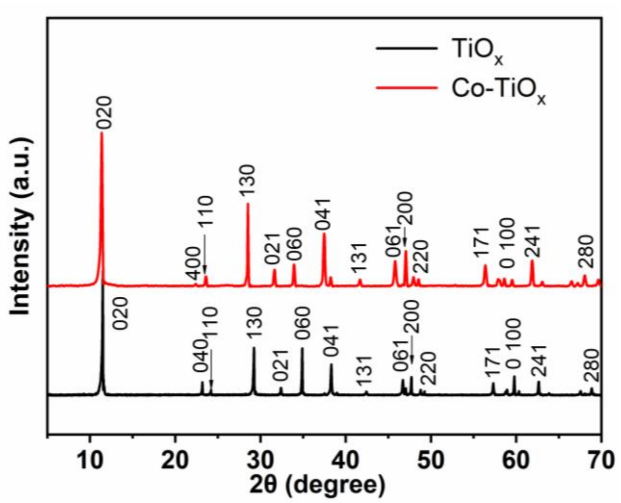

Fig. S6. XRD analysis. XRD patterns of parent layered TiO_x and Co-TiO_x powders.

The X-ray diffraction (XRD) patterns of parent layered TiO_x and Co-TiO_x demonstrate sharp diffraction peaks, indicating high crystallinities. The typical diffraction peaks located at 2θ = 11.3 °, 22.5 °, 23.6 °, 28.5 °, 31.7 °, 33.9, 37.5, 41.7 °, 45.8 °, 47.0 °, 48.6, 56.4 °, 58.7, 61.9 and 68.1 ° for Co-TiO_x bulk represent the (020), (400), (110), (130), (021), (060), (041), (131), (061), (200), (220), (171), (0100), (241) and (280) reflections of a lepidocrocite-type crystal structure.

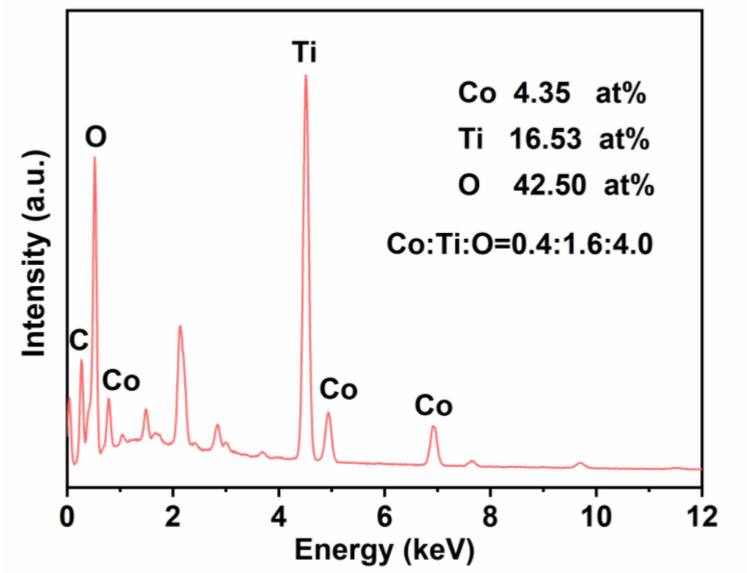

Fig. S7. EDX analysis. Energy dispersive X-ray spectroscopy (EDX) of Co-TiO_x free standing membrane.

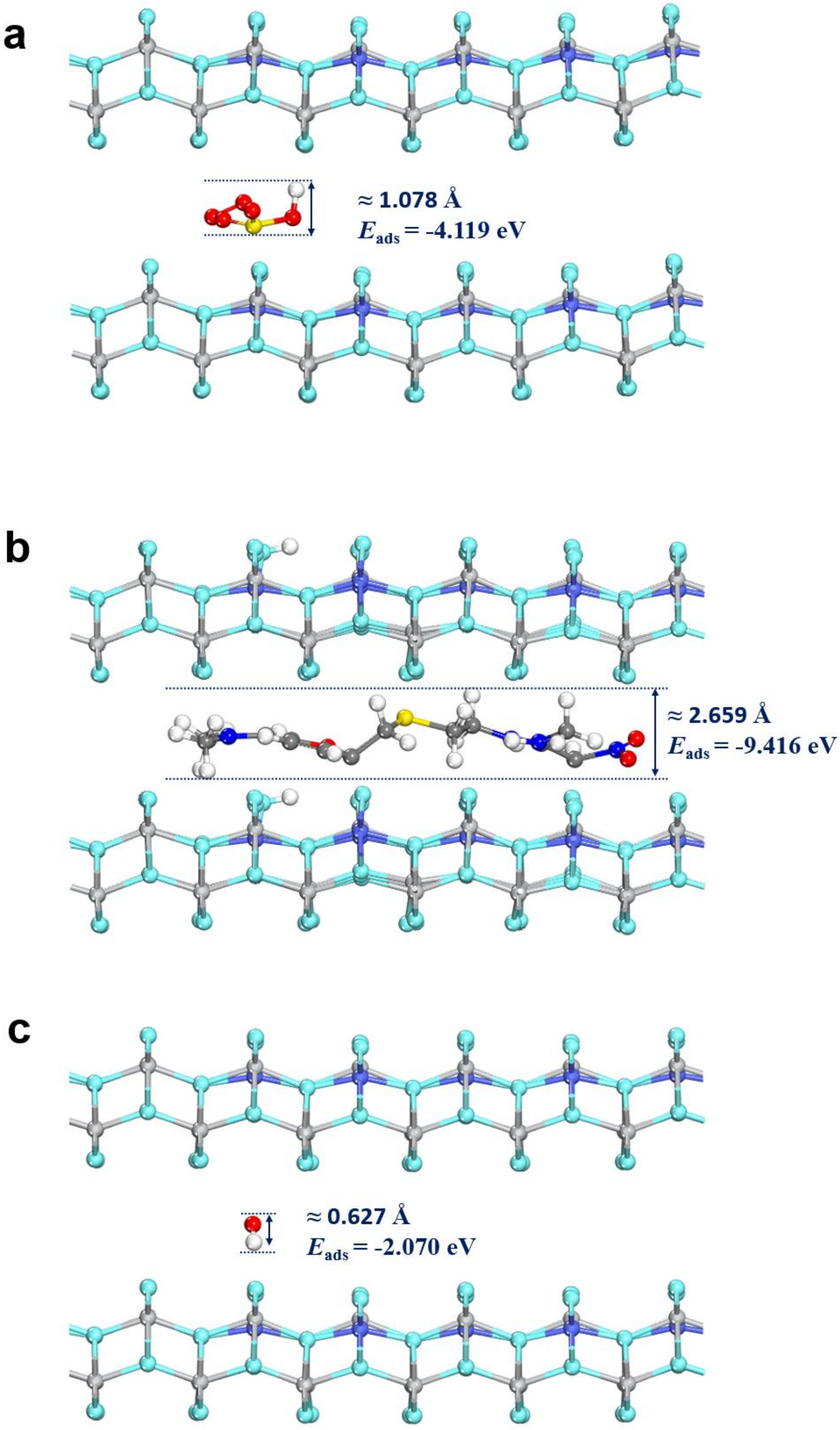

Fig. S8. Molecular size and adsorption energy analysis. Molecular size and adsorption energy for PMS (a), ranitidine (b) and water (c) intercalated into Co-TiO_x nanosheets.

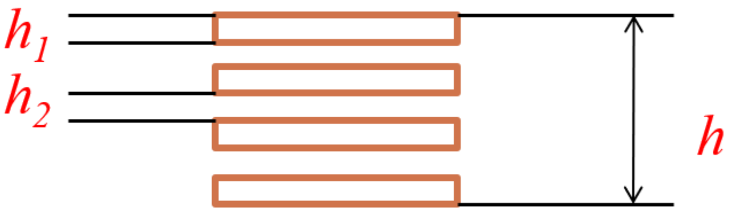

Fig. S9. Retention time calculation. Calculation of retention time of the Co-TiO$_x$ membrane.

The membrane was treated as a kind of reaction cell with a uniform thickness and interlayer spacing of Co-TiO$_x$ nanosheets. The retention time was calculated using the following equations:

$$h = nh_1 + (n-1)h_2 \tag{1}$$

$$h' = (n-1)h_2 \tag{2}$$

$$h' = \left(\frac{h - h_1}{h_1 + h_2}\right)h_2 \approx \frac{hh_2}{h_1 + h_2} \tag{3}$$

$$t = \frac{V}{Q} = \frac{S \times h'}{J \times S \times P} = \frac{h'}{J \times P} \tag{4}$$

Where V is the inner volume of membrane (cm^3), Q is the flow rate (mL/min). h, h_1, and h_2 (nm) are the thickness of Co-TiO$_x$ membrane, Co-TiO$_x$ nanosheet, and interlayer free spacing, respectively. h' (nm) is the integral of h_2. S (m^2) is the effective membrane filtration area. The values of h (500 nm), h_1 (0.69 nm), and h_2 (0.46 nm) are obtained from the cross-sectional SEM, TEM, and XRD results, respectively. J (L m^{-2} h^{-1} bar^{-1}) and P (bar) are obtained from experiments.

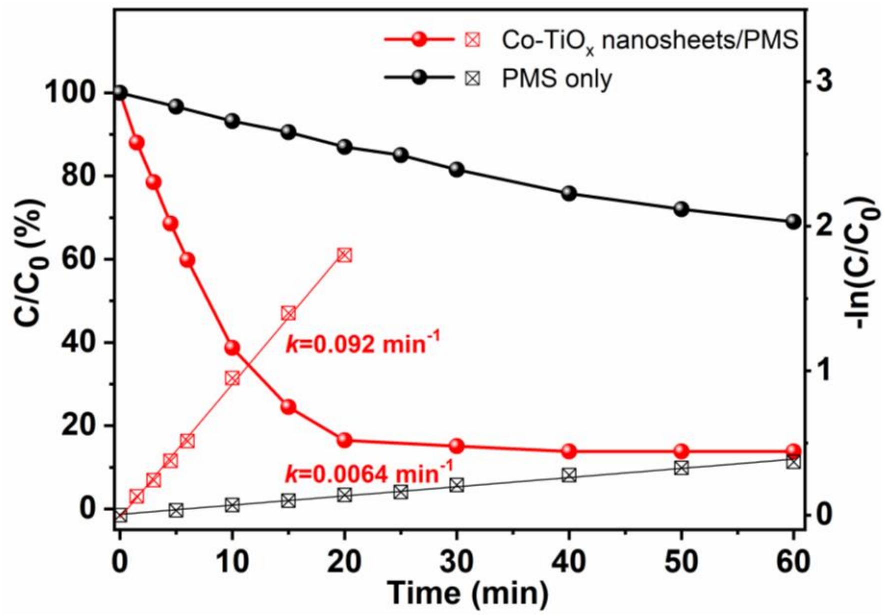

Fig. S10. Degradation kinetics. Degradation and first-order reaction kinetics of ranitidine removal by PMS and Co-TiO$_x$ nanosheets/PMS.

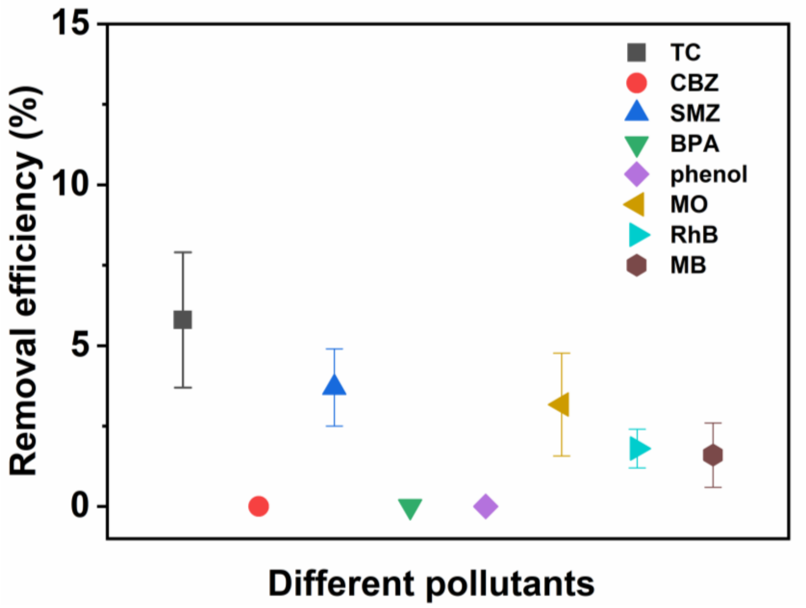

Fig. S11. Removal efficiency by PMS. The removal efficiency of different pollutants in PMS system without catalysts.

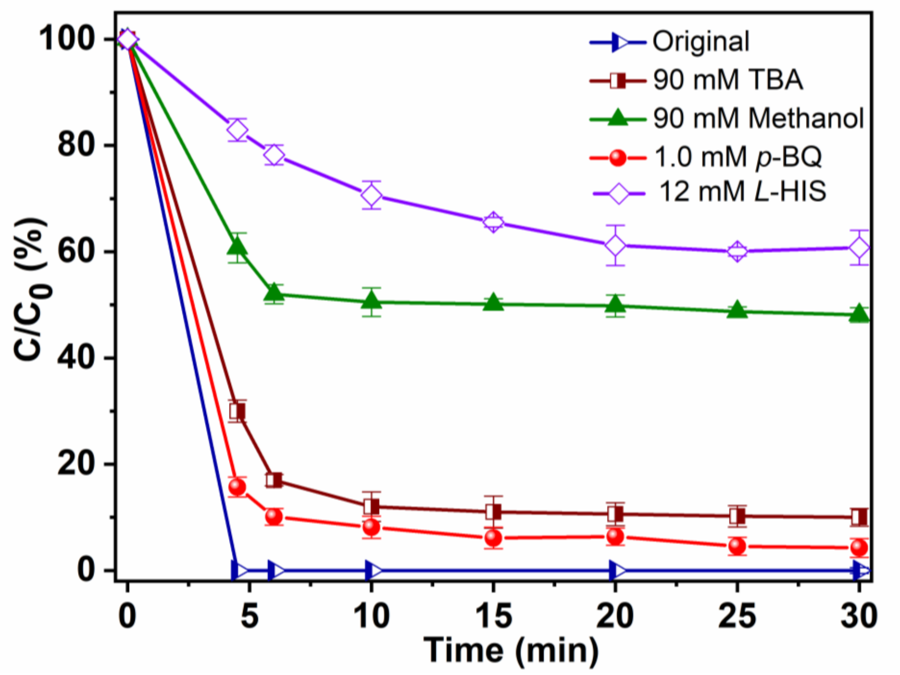

Fig. S12. Quenching experiments. Inhibition effect of radical scavengers on ranitidine degradation in the Co-TiO$_x$ membrane/PMS system.

As shown in Fig. S12, the contribution of reactive species was investigated *via* quenching experiments, and the quenching agents include methanol, TBA, *p*-BQ, and *L*-HIS. (1) The removal efficiency of ranitidine slightly decreases in the presence of TBA (scavenger for $^{\bullet}OH$), indicating that $^{\bullet}OH$ participates in the degradation reaction. (2) After the addition of methanol (scavenger for $^{\bullet}OH$ and $SO_4^{\bullet-}$), degradation efficiency shows a further decrease, indicating the participation of $SO_4^{\bullet-}$ in the reaction and the bigger contribution of $SO_4^{\bullet-}$ compared to $^{\bullet}OH$. (3) The removal efficiency slightly decreases after the addition of *p*-BQ (scavenger for $^{\bullet}O_2^-$). The slight decrease could be attributed to the covering of the active sites of catalysts by the adsorbed *p*-benzoquinone. (4) Notably, when *L*-HIS, a sacrificial agent for singlet oxygen ($^1O_2$) was added into the reaction system, the degradation of ranitidine was dramatically inhibited.

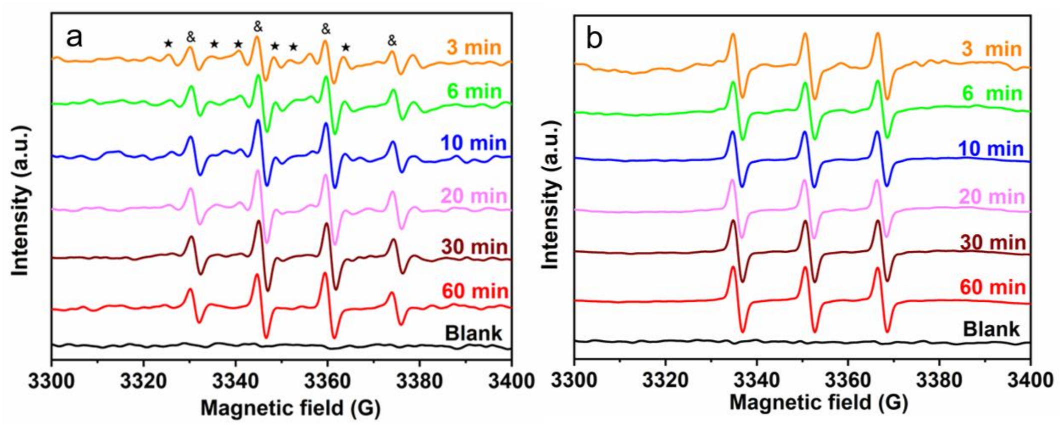

Fig. S13. Identification of the dominant active species. EPR spectra of (a) $^{\bullet}OH$ and $SO_4^{\bullet-}$ and (b) $^1O_2$ obtained from the Co-TiO$_x$ membrane/PMS system. ★ and & represent $SO_4^{\bullet-}$ and $^{\bullet}OH$, respectively.

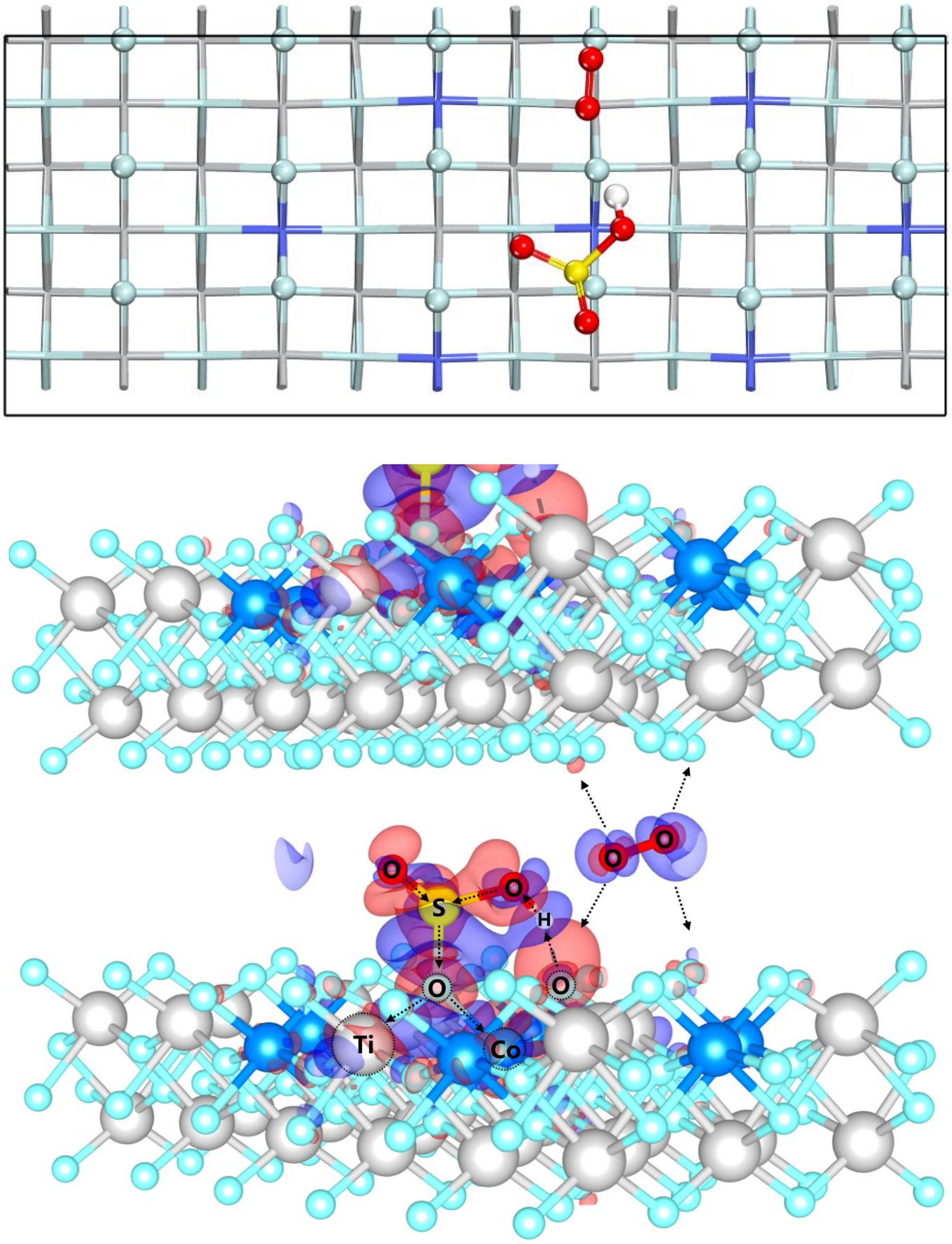

Fig. S14. EDD analysis. Electronic density difference for PMS intercalating into Co-TiO$_x$ nanosheets; red and blue represent electron accumulation and depletion, respectively.

The electron density difference (EDD) analysis was performed to understand the changes of electron density distribution between PMS and Co-TiO$_x$ nanosheets. The EDD was defined as

$$\Delta\rho(r) = \rho_{\mathrm{system}}(\mathrm{r}) - \rho_{\mathrm{surface}}(\mathrm{r}) - \rho_{\mathrm{HSO_3^-}}(\mathrm{r}) - \rho_{^1\mathrm{O_2}}(\mathrm{r}) \qquad (5)$$

where ρ_{system}, ρ_{surface}, $\rho_{\mathrm{HSO3-}}$, and ρ_{1O2} are the electronic densities of the PMS/Co-TiO$_x$,

Co-TiO$_x$, HSO_3^-, and $^1O_2$ species, respectively. 3D colored EDD image is presented in Fig. S14, where the red and blue isosurfaces represent electronic accumulation and depletion, respectively. The large redistributions of electronic densities are observed between S atom of HSO_3^- and O atom of the substrate (S–>O). It indicates that the interactions between HSO_3^- and Co-TiO$_x$ nanosheets are rather strong mainly due to their electrostatic characteristics. Notably, the electronic redistributions are also obtained between Co atom and O atom in Co-TiO$_x$ nanosheets (O–>Co), which denotes that the substituted Co atoms in Co-TiO$_x$ nanosheets promote PMS activation.

The generation of reactive oxygen species including $^{\bullet}OH$, $SO_4^{\bullet-}$ and $^1O_2$ can be illustrated as follows:

$$\mathbf{HSO_5^- \rightarrow {}^1O_2 + HSO_3^-} \quad \mathbf{(6)}$$

$$\mathbf{\equiv Co\ (II) + HSO_5^- \rightarrow \equiv Co\ (III) + SO_4^{\bullet-} + OH^-} \quad \mathbf{(7)}$$

$$\mathbf{SO_4^{\bullet-} + H_2O \rightarrow SO_4^{2-} + {}^{\bullet}OH + H^+} \quad \mathbf{(8)}$$

$$\mathbf{\equiv Co\ (III) + HSO_5^- \rightarrow \equiv Co\ (II) + SO_5^{\bullet-} + H^+} \quad \mathbf{(9)}$$

$$\mathbf{{}^1O_2/\ SO_4^{\bullet-}/\ {}^{\bullet}OH + ranitidine/pollutants \rightarrow degraded\ products} \quad \mathbf{(10)}$$

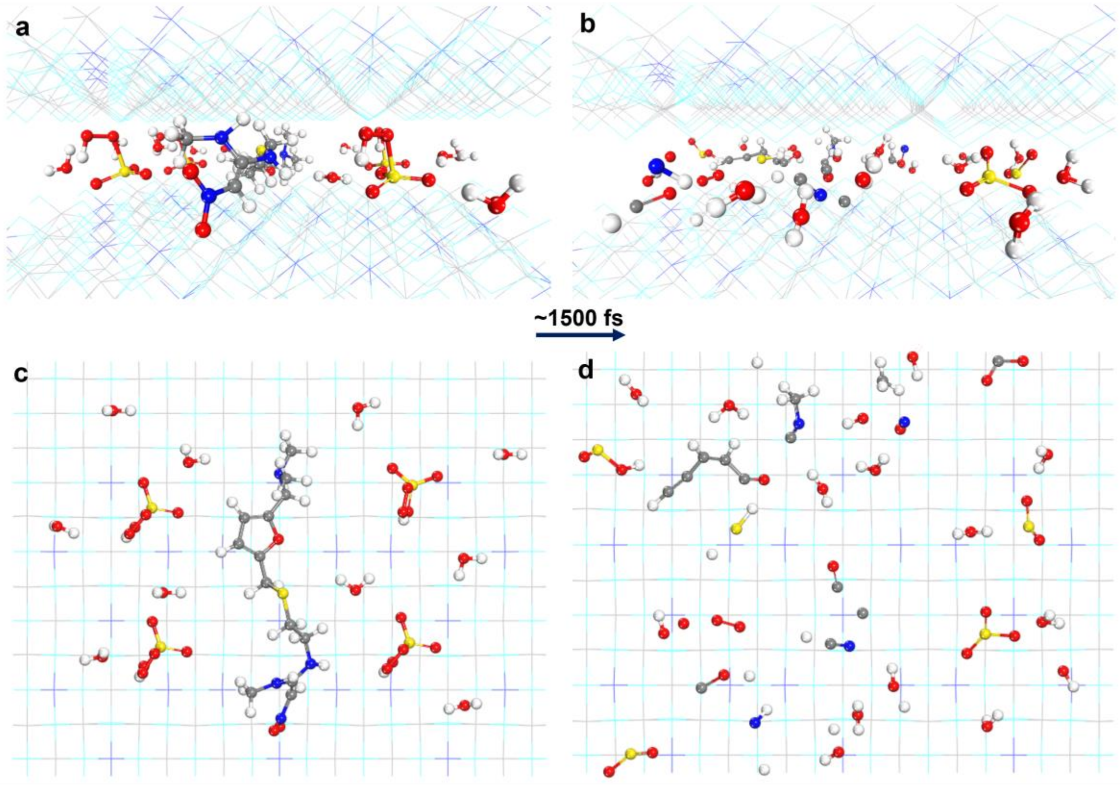

Fig. S15. AIMD simulations of molecules within Co-TiO$_x$ nanochannels. AIMD simulations (a and c: initial state; b and d: final state after 1500 fs) of the diffusion of PMS, ranitidine, and H_2O molecules inside Co-TiO$_x$ nanochannels with the interlayer free spacing of 0.46 nm.

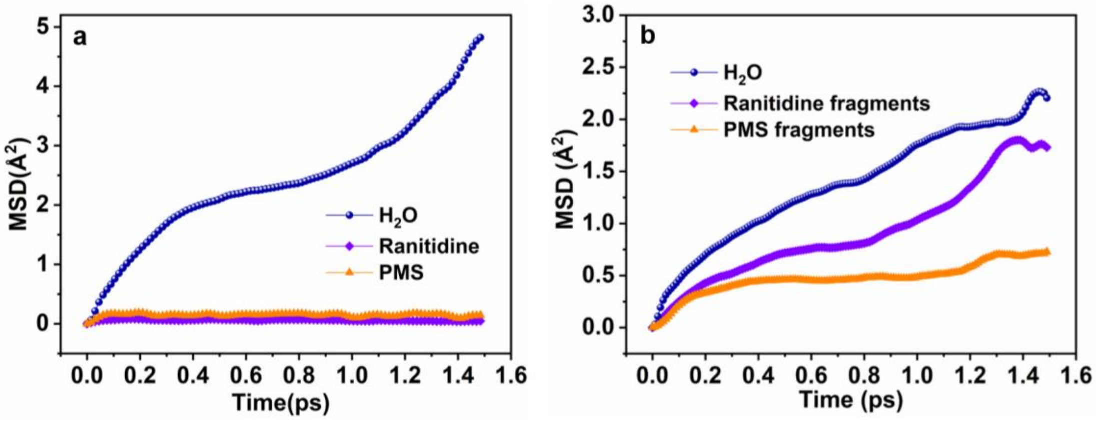

Fig. S16. The diffusion performance of molecules within Co-TiO$_x$ nanochannels. MSD curves of PMS, ranitidine, and H_2O molecules (a) and PMS fragments, ranitidine fragments, and H_2O molecules (b) inside Co-TiO$_x$ nanochannels with the

interlayer free spacing of 0.46 nm.

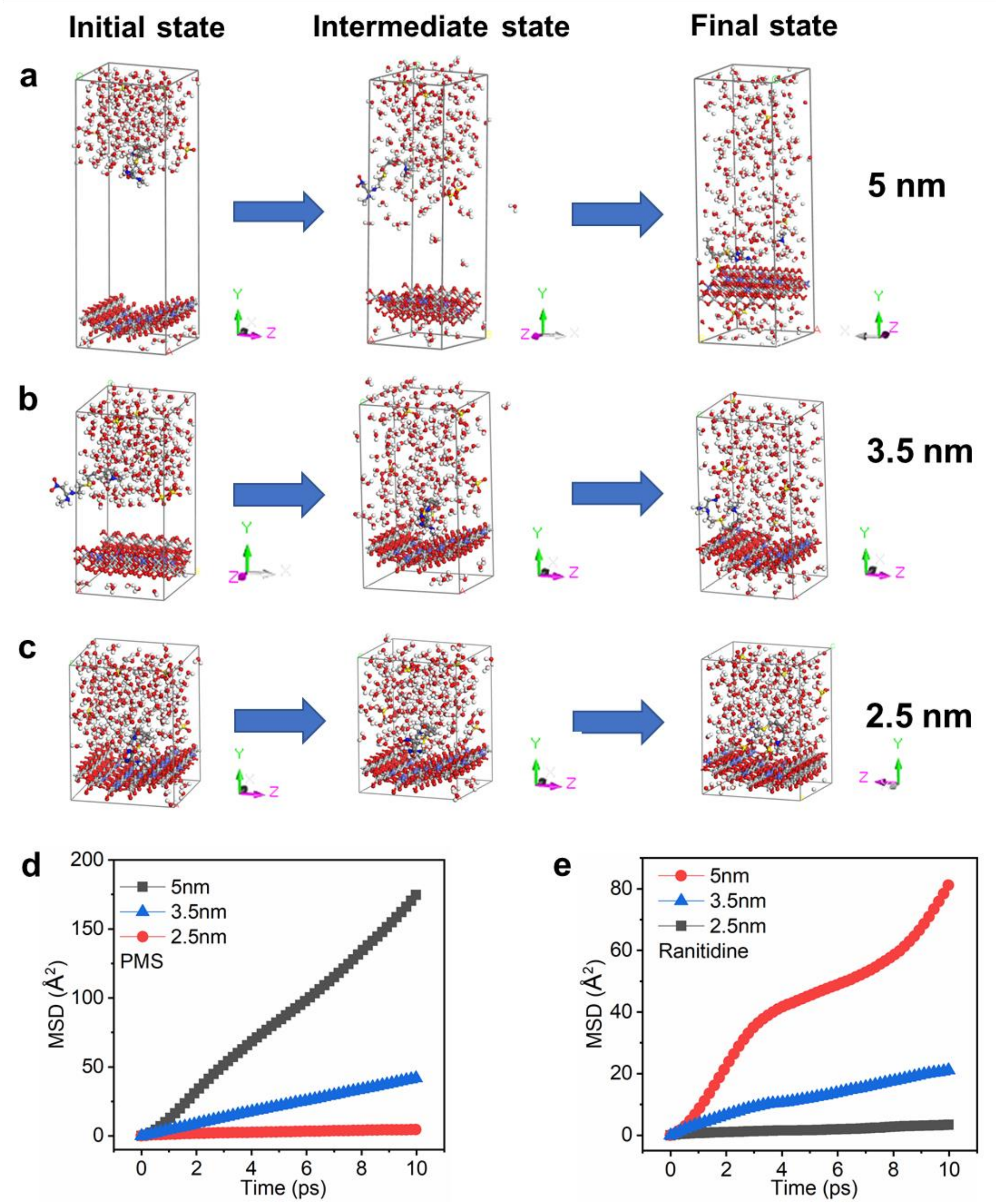

Fig. S17. The diffusion process of PMS, ranitidine, and water within Co-TiO$_x$ nanochannels. MD simulations of the diffusion of PMS, ranitidine, and H_2O molecules (a-c) and the MSD of PMS molecules (d) and ranitidine molecules (e) inside Co-TiO$_x$ nanochannels with three different interlayer free spacings (2.5, 3.5, 5 nm).

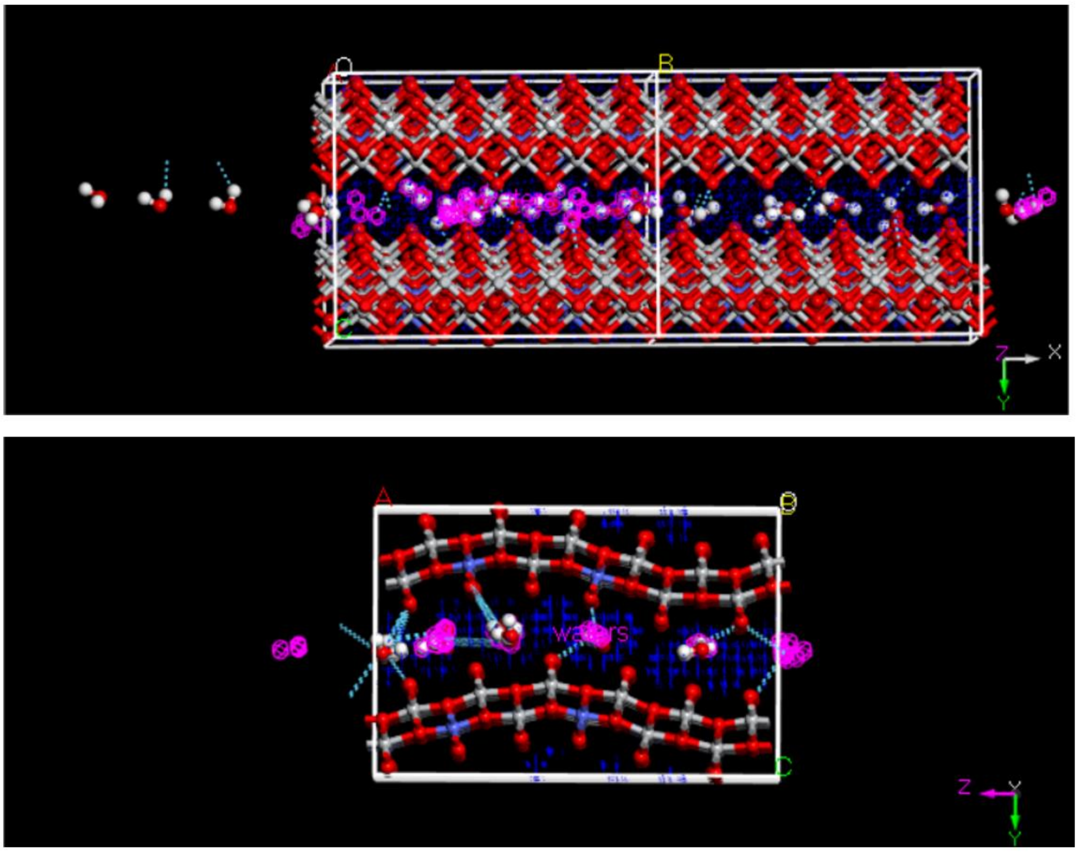

Fig. S18. The diffusion process of water and hydrogen bonding within Co-TiO$_x$ nanochannels. The transportation of water molecules within Co-TiO$_x$ nanosheets and hydrogen bonding interaction between water molecules and Co-TiO$_x$ nanosheets with the interlayer free spacing of 0.46 nm.

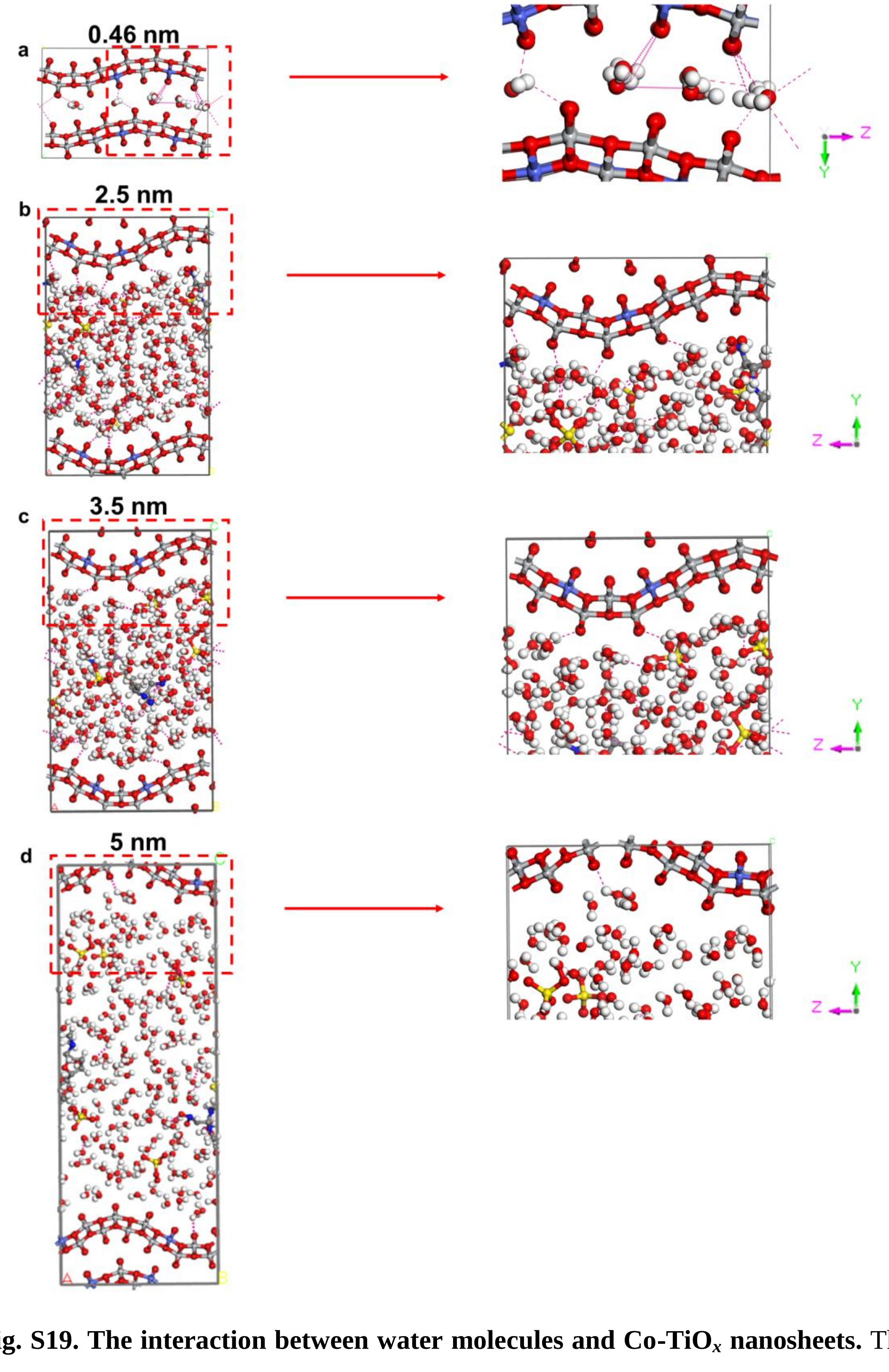

Fig. S19. The interaction between water molecules and Co-TiO$_x$ nanosheets. The hydrogen bonding interaction between water molecules and Co-TiO$_x$ nanosheets with different interlayer free spacings (a-0.46 nm, b-2.5 nm, c-3.5 nm, d-5 nm).

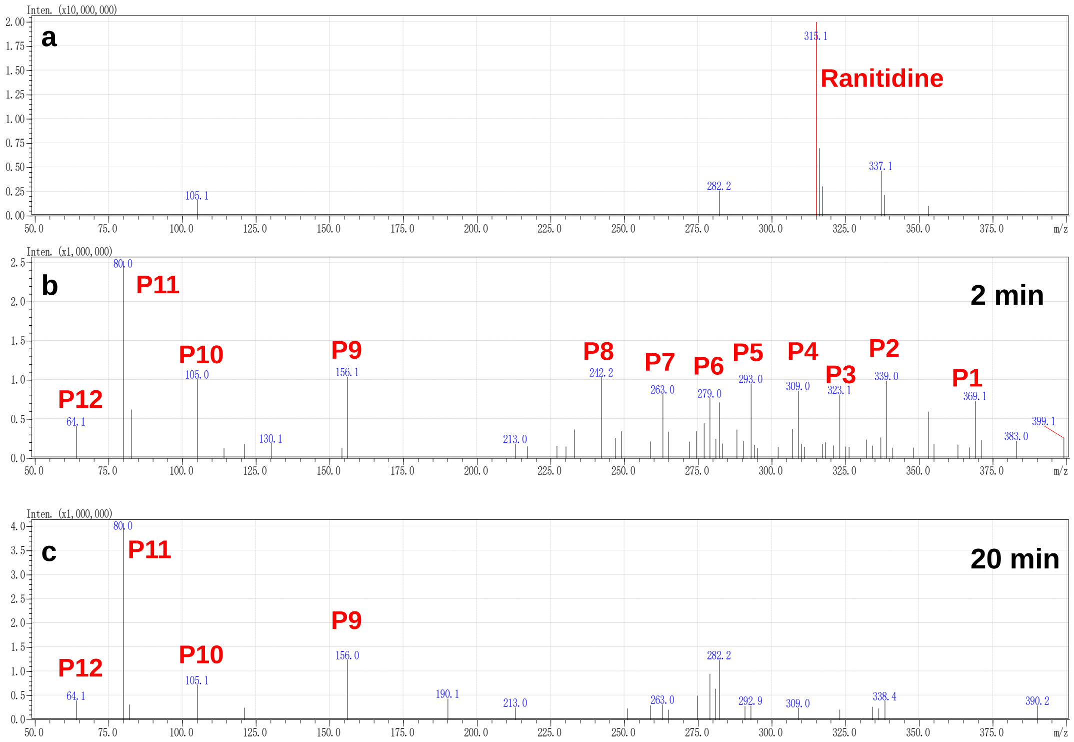

Fig. S20. The degradation products of ranitidine. Spectra of ranitidine degradation products obtained in ESI(+)-MS mode by LC-MS. (a) feed solution (t = 0 min); permeate solutions (b) (t = 2 min) and (c) (t = 20 min).

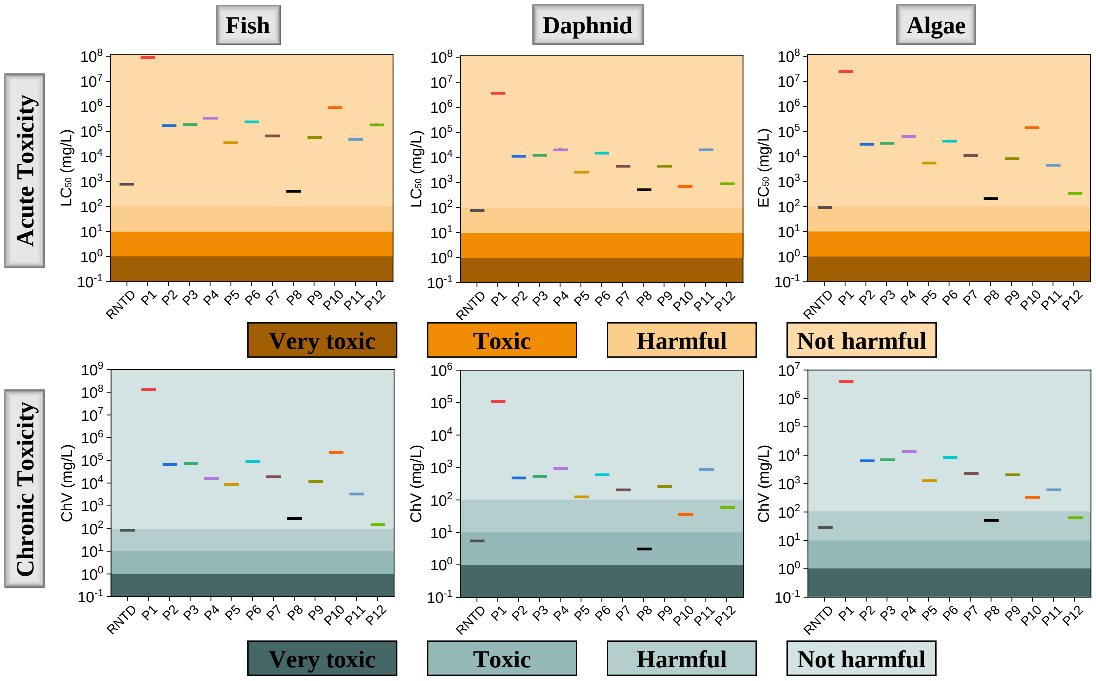

Fig. S21. Toxicity estimation of ranitidine and its degradation intermediates using the ECOSAR program. According to the system established by the Globally Harmonized System of Classification and Labeling of Chemicals (GHS), the predicted toxicity values of ranitidine and all intermediates can be divided into four categories: very toxic ($LC_{50}/EC_{50}/ChV < 1$ mg/L), toxic (1 mg/L $< LC_{50}/EC_{50}/ChV <$ 10 mg/L), harmful (10 mg/L $< LC_{50}/EC_{50}/ChV <$ 100 mg/L), and not harmful ($LC_{50}/EC_{50}/ChV >$ 100 mg/L)[2].

Supplementary Tables

Table S1. Comparison of ranitidine removal efficiency over different technologies.

No.	Methods	Materials	Ranitidine concentration (mg/L)	Ranitidine removal efficiency	Reaction time	Catalysts dosage	k (min^{-1})	Ref
1	Photocatalysis	MoS_2/RGO	10	74%	60 min	1 g/L	0.0208	3
2	Photocatalysis	MoS_2	10	33%	60 min	1 g/L	0.00599	3
3	Photocatalysis	RGO	10	35%	60 min	1 g/L	0.00644	3
4	Photocatalysis	MXene-Ti_3C_2/MoS_2	10	88.4%	60 min	1 g/L	0.03148	4
5	Photocatalysis	MXene-Ti_3C_2	10	18.4%	60 min	1 g/L	0.0032	4
6	Photocatalysis	TiO_2	10	100%	45 min	0.2 g/L	0.146	4
7	Photo-Fenton	TiO_2+Fe^{2+}/H_2O_2	10	100%	22 min	0.2 g/L	0.23	5
8	Photocatalysis	TiO_2 Nanofiber	3	95%	120 min	--	0.0080	6
9	Photocatalysis	Degussa P25 naoparticles	3	96%	120 min	--	0.011	6
10	Photocatalysis	TiO_2	50	60%	5 h	1 g/L	0.00396	7
11	UV photolysis	NH_2Cl	5	89.4%	5 min	0.051-0.3 g/L	0.33	8
12	Heterogeneous Fenton-like catalysis	nZVIPs@Ti_3C_2 nanosheets	5	92.7%	30 min	0.1 g/L	0.0721	9
13	Heterogeneous catalysis	Co-TiO_x nanosheets	5	85.4%	20 min	0.017 g/L	0.092	This work
14	Nanoconfinement catalysis	Co-TiO_x membrane	5	100%	5.5 ms	0.08 mg/cm^2	63600	This work

Table S2. Operating conditions and the result of ICP-MS.

**Operating conditions**	RF Power	1.5 kW	Plasma flow	15.0 L/min
	Auxiliary flow	1.0 L/min	Nebulizer flow	0.10 L/min
	Sample untake delay	30 s	Instr stabilization delay	30 s
	Replicate read time	5 s	Replicates	3 times
**Result**	Element	Co	Concentration	0. 2 μg/L

Table S3. XPS spectra of Co-TiO$_x$ membranes before and after stability test.

**Element**	**Bond type**	**Before stability test**				**After stability test**			
		**E/eV**	**at/%**	**Peak area**	**FWHM**	**E/eV**	**at/%**	**Peak area**	**FWHM**
Co 2*p*	≡Co(II)	781.0	31.63	6233.67	3.44	781.8	13.07	1939.28	3.50
		786.0	18.96	3716.88	4.25	786.3	20.65	3049.23	3.52
		796.8	10.16	1970.42	2.41	796.3	16.10	2352.71	2.56
		802.2	14.79	2850.11	3.51	802.5	14.73	2138.69	3.56
	≡Co(III)	779.7	15.08	2976.37	1.59	780.0	31.09	4621.44	2.11
		795.3	9.37	1819.27	1.89	795.1	4.35	636.70	1.50
O 1*s* (at %)		47.3				44.2			
C 1*s* (at %)		26.0				27.9			
Ti 2*p* (at %)		19.4				19.1			
Co 2*p* (at %)		5.6				5.1			
N 1*s* (at %)		3.8				3.7			

Table S4. Operating conditions of LC-MS for micropollutant quantification.

**LC operating conditions**		**MS operating conditions**	
Eluent A	0.05% Formic acid	Detection mode and temperature	ESI and 300℃
Eluent B	Methanol	Heating block temperature	400℃
Column temperature	40℃	Atomization flow rate	2.5 L/min
Sample injection	10 μL	Gas flow rate	10 L/min
		DL temperature	250℃

Table S5. Degradation products of ranitidine in the Co-TiO$_x$ membrane/PMS system.

Compounds	m/z value	Structure
Ranitidine	315	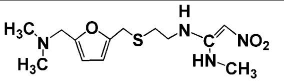
P1	369	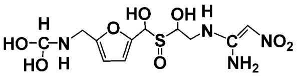
P2	339	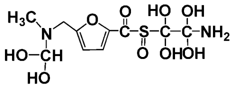
P3	323	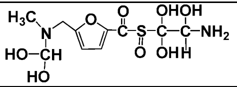
P4	309	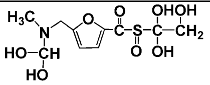
P5	293	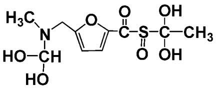
P6	279	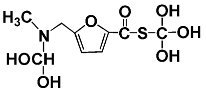
P7	263	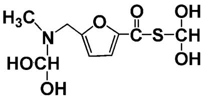
P8	242	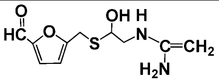
P9	156	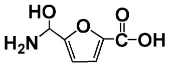
P10	105	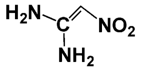
P11	80	$HOH_2C-S-OH$
P12	64	$H_3C-S-OH$

Supplementary References

1 Dou, Y. et al. TiO_2@layered double hydroxide core-shell nanospheres with largely enhanced photocatalytic activity toward O_2 generation. *Adv. Funct. Mater.* **25**, 2243–2249 (2015). DOI: 10.1002/adfm.201404496

2 Wang, J. B., Zhi, D., Zhou, H., He, X. W., Zhang, D. Y. Evaluating tetracycline degradation pathway and intermediate toxicity during the electrochemical oxidation over a Ti/Ti_4O_7 anode, *Water Res.* **137** 324–334 (2018). DOI: 10.1016/j.watres.2018.03.030

3 Zou, X., Zhang, J., Zhao, X. & Zhang, Z. MoS_2/RGO composites for photocatalytic degradation of ranitidine and elimination of NDMA formation potential under visible light. *Chem. Eng. J.*, **383**, 123084 (2020). DOI: 10.1016/j.cej.2019.123084

4 Zou, X., Zhao, X., Zhang, J., Lv, W., Qiu, L. & Zhang, Z. Photocatalytic degradation of ranitidine and reduction of nitrosamine dimethylamine formation potential over MXene-$Ti3C_2/MoS_2$ under visible light irradiation. *J. Hazard. Mater.* **413**, 125424 (2021). DOI: 10.1016/j.jhazmat.2021.125424

5 Radjenovic, J., Sirtori, C., Petrovic, M., Barcelo, D. & Malato, S. Characterization of intermediate products of solar photocatalytic degradation of ranitidine at pilot-scale. *Chemosphere* **79**, 368–376 (2010). DOI: 10.1016/j.chemosphere.2010.02.014

6 Choi, K. J., Hong, S. W. Preparation of TiO_2 nanofibers immobilized on quartz substrate by electrospinning for photocatalytic degradation of ranitidine. *Res. Chem. Intermed.* **38**, 1161–1169 (2012). DOI: 10.1007/s11164-011-0455-z

7 Addamo, M., Augugliaro, V., Paola, A. D., García-López, E., Loddo, V., Marcì, G. & Palmisano, L. Removal of drugs in aqueous systems by photoassisted degradation. *J. Appl. Electrochem.* **35**, 765–774 (2005). DOI: 10.1007/s10800-005-1630-y

8 Wu, Y., Zhu, S., Wang, J., Bu, L., Deng, J. & Zhou, S. Role of reactive nitrogen species in ranitidine degradation in UV/chloramine process: transformation pathways and NDMA formation. *Chem. Eng. J.* **404**, 126557 (2021). DOI:

10.1016/j.cej.2020.126557

9 Ma, Y., Lv, X., Xiong, D., Zhao, X. & Zhang, Z. Catalytic degradation of ranitidine using novel magnetic Ti_3C_2-based MXene nanosheets modified with nanoscale zero-valent iron particles. *Appl. Catal. B. Environ.* **284**, 119720−119733 (2021). DOI: 10.1016/j.apcatb.2020.119720
